# Double Pedicle Approach for the Management of Gingival Recession in Severely Crowded Teeth: A Case Report

**DOI:** 10.7759/cureus.78876

**Published:** 2025-02-11

**Authors:** Shivlal Vishnoi, Meghna Gohil, Pooja Challapali, Priyadarshini Nadig, Dhwani Bharucha

**Affiliations:** 1 Department of Periodontology, Manubhai Patel Dental College and Hospital, Vadodara, IND

**Keywords:** attachment loss, double pedicle technique, gingival recession, periodontal plastic surgeries, root coverage

## Abstract

Contemporary patients increasingly prioritize aesthetic considerations in their dental care. Consequently, esthetic considerations have become an essential component of periodontal treatment planning and delivery. Several techniques have been proposed for the coverage of gingival recession; however, a comprehensive understanding of the various conditions of denuded root surfaces is essential for achieving predictable outcomes in root coverage. When aesthetic demand is high, where the papillae are large and have shallow gingival grooves, a double pedicle flap can serve as a potential treatment alternative. This case report describes the effective management of a female patient presenting with dentinal hypersensitivity and receding gums, which impaired her ability to maintain proper oral hygiene and consume hot or cold foods. A double pedicle flap approach was used to treat the denuded root surface, leading to an increase in keratinized tissue, alleviation of dentinal hypersensitivity, and acceptable root coverage.

## Introduction

Gingival recession is defined as the displacement of the gingival margin apical to the cementoenamel junction [1]. Periodontal disease and poor oral hygiene habits are the main etiological factors causing gingival recession. Additional predisposing conditions such as gingival biotype, tooth morphology, dental alignment, frenum attachment, and alveolar bone dehiscence can also increase the risk of recession.

Pedicle graft procedures can be classified based on the direction of tissue transfer into two categories: (i) rotational flaps, which include lateral sliding flaps and papilla flaps; and (ii) advanced flaps, which may involve movement with or without rotation or lateral displacement [2]. The double papilla flap procedure was initially introduced by Wainberg as the double lateral repositioned flap and later refined by Cohen and Ross, who coined the term "double papilla flap" [3].

The success of root coverage procedures is significantly influenced by the interdental bone height and the adjacent soft tissue morphology. Additionally, the defect's dimensions, the extent of avascular tooth surface exposed to the graft during initial healing, and the quantity of graft material employed all play pivotal roles in determining the ultimate outcome.

## Case presentation

**Figure 1:**
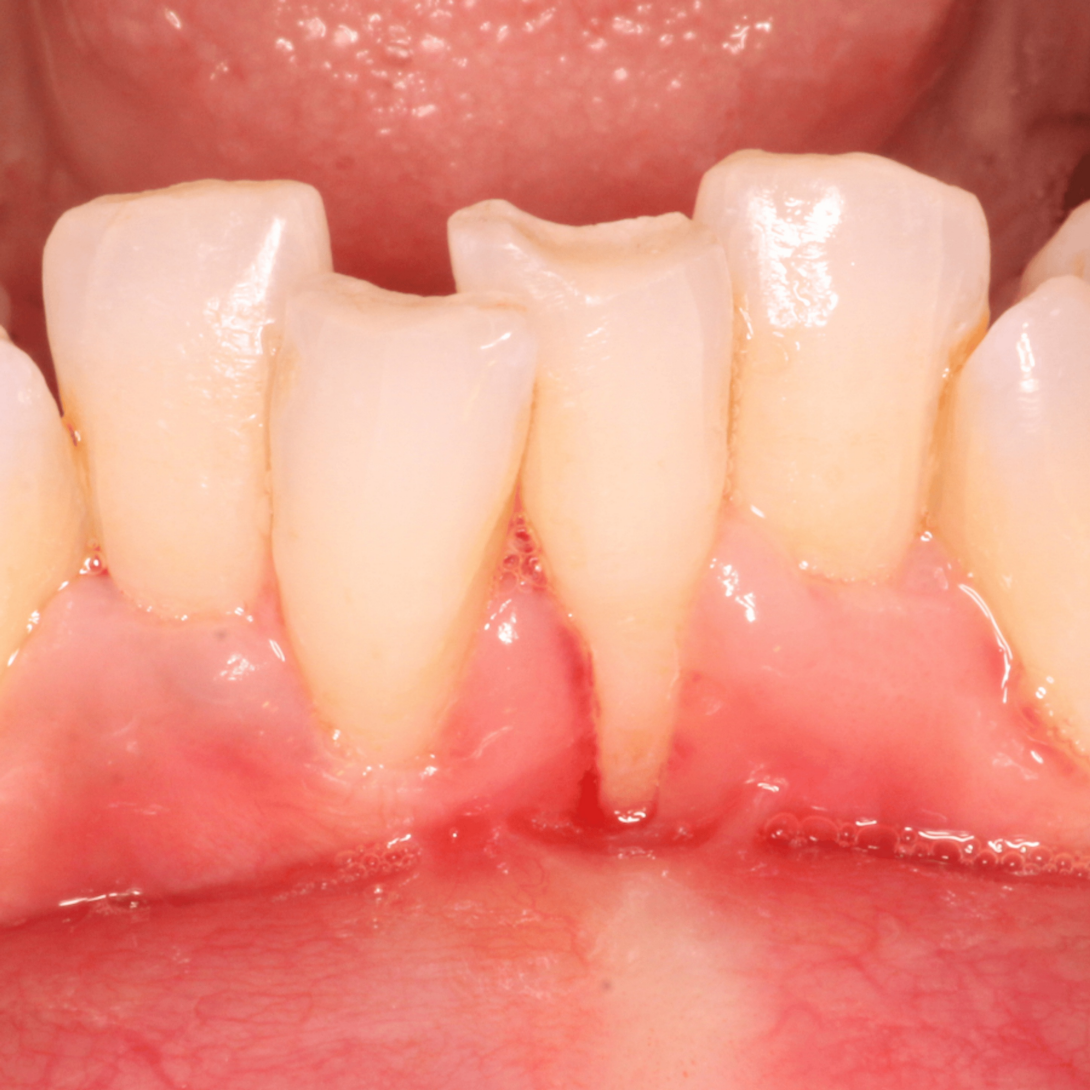
Preoperative site with gingival recession i.r.t 31, 41

**Figure 2:**
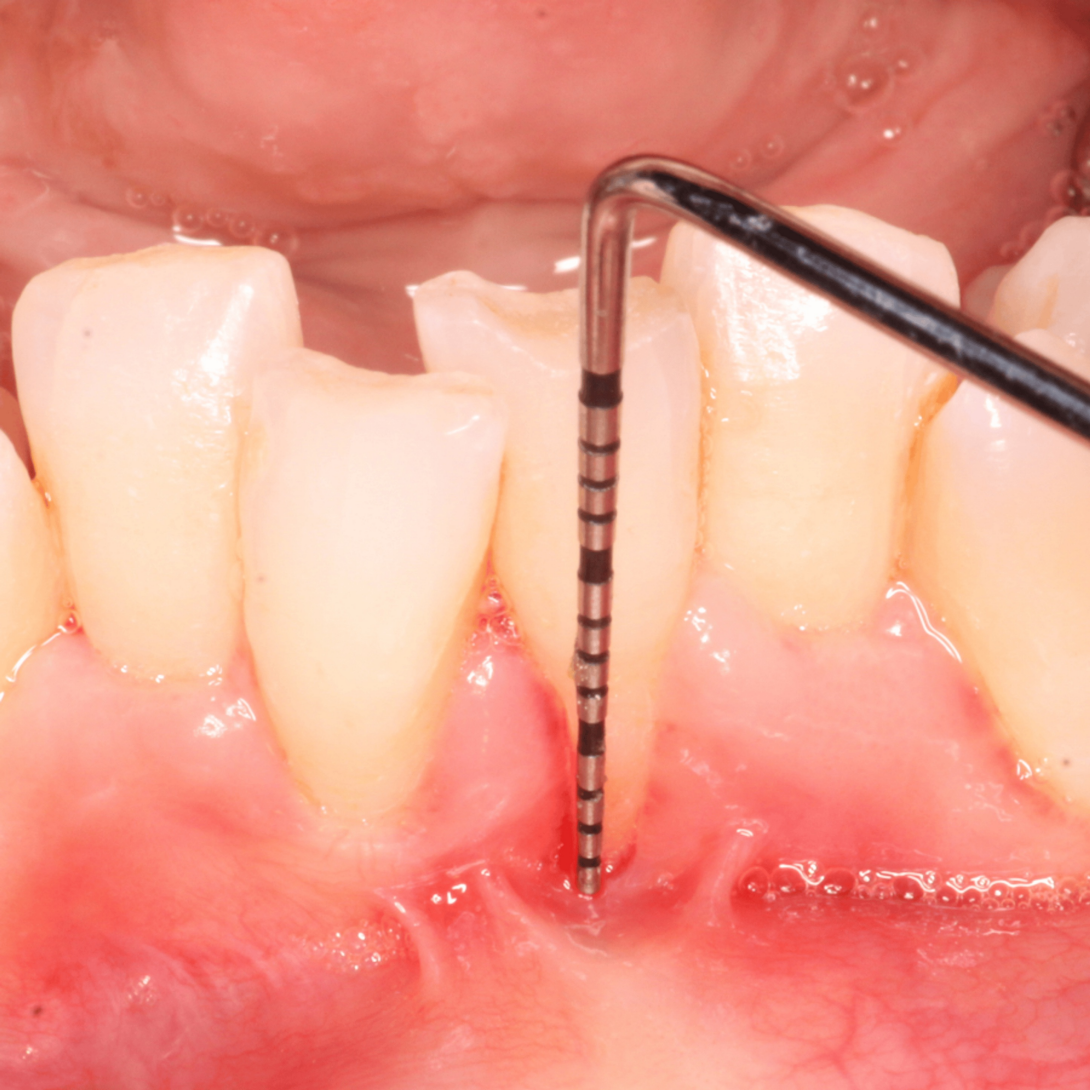
Miller’s class II gingival recession (5 mm) along with minimal vestibular depth

A 35-year-old female patient was referred to the Department of Periodontics and Oral Implantology, with the chief complaint of gum recession and dentinal hypersensitivity in lower central incisors (i.e., 31, 41). The patient was a nonsmoker and in good general health and had not received any antibiotics or periodontal therapy in the last six months. On intraoral examination, a Miller class II gingival recession along with minimal vestibular depth was seen. Although there was no trauma from occlusion, the teeth were severely crowded in the lower anterior. A thorough phase 1 therapy was done followed by the current surgical treatment modality planned, i.e., double pedicle flap. Prior to the therapy, a clinical preoperative photograph (Figure 1) with clinical measurements was taken, with a probing depth of 1 mm, gingival recession of 5 mm, and clinical attachment level of 5 mm using the University of North Carolina (UNC)-15 periodontal probe (Figure 2).

Presurgical phase

The patient received comprehensive education regarding the procedure, provided informed consent, and was instructed on proper oral hygiene techniques, with particular emphasis on brushing habits. A thorough debridement was performed, including scaling and root planing. Regular follow-up appointments were scheduled to monitor oral hygiene and gingival health.

Surgical phase

**Figure 3:**
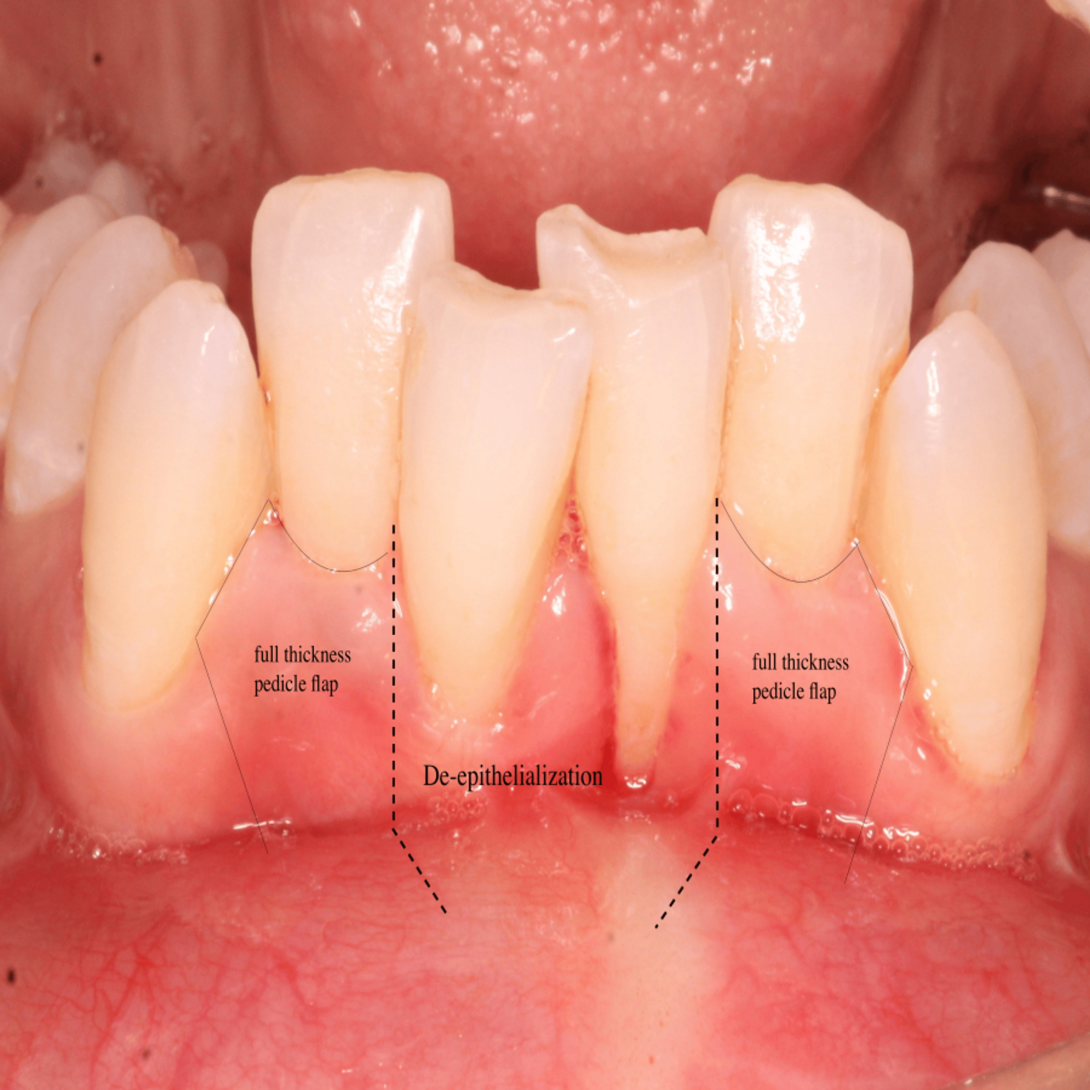
Proposed flap illustration for double pedicle flap

**Figure 4:**
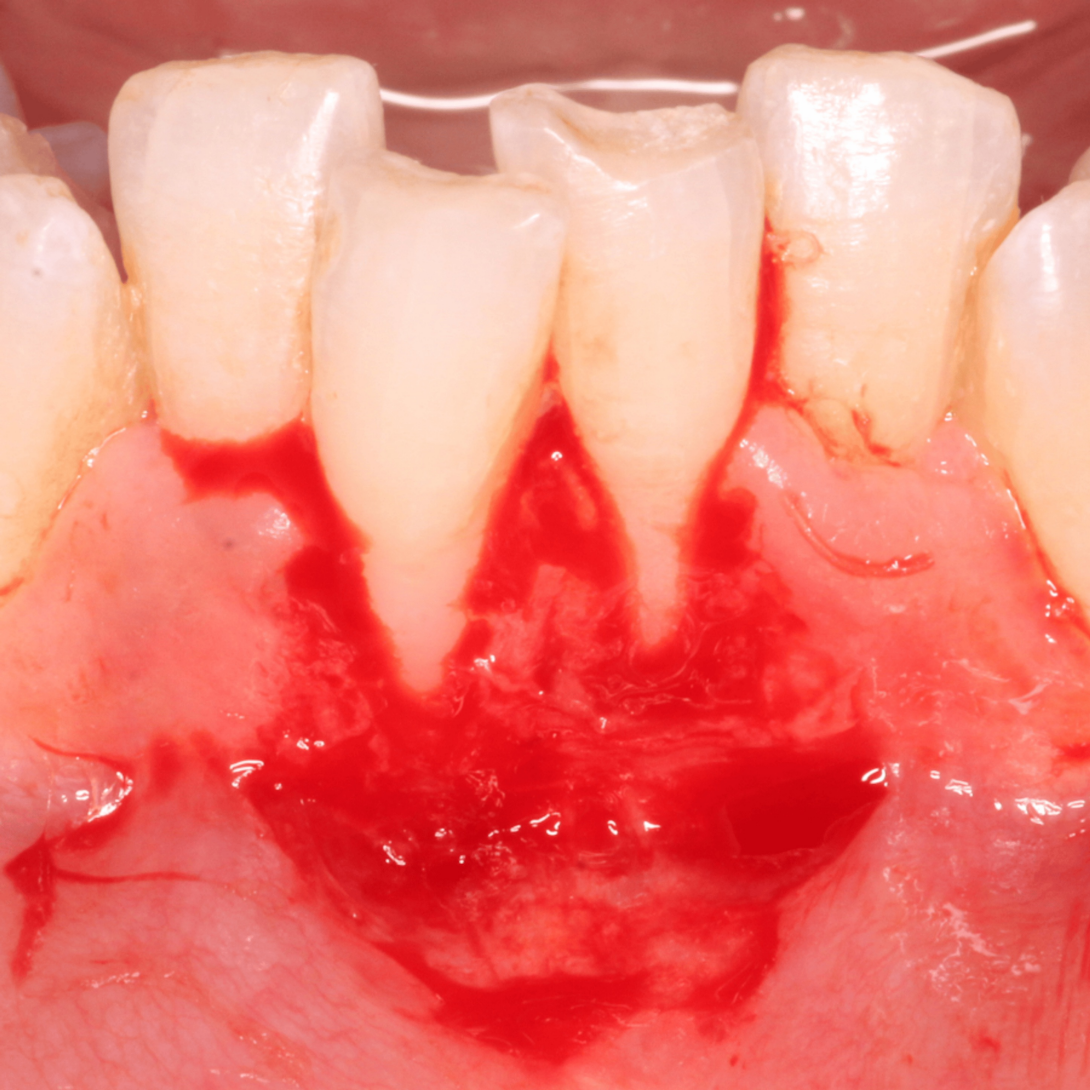
Deepithelialization of recipient site with two vertical releasing incisions

**Figure 5:**
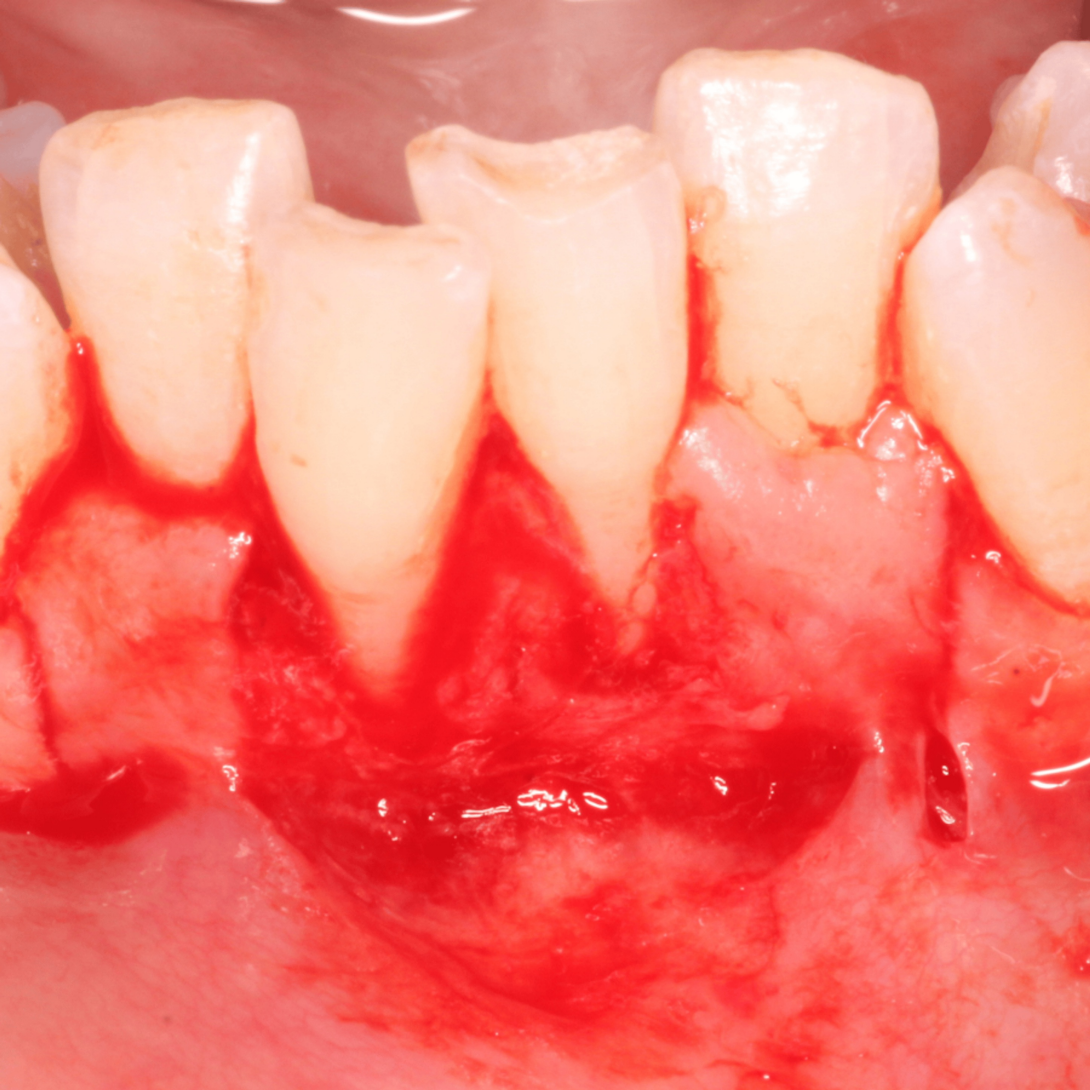
Intracrevicular incision followed by two vertical incisions extending beyond MGJ at the line angles of the adjacent teeth MGJ: mucogingival junction

**Figure 6:**
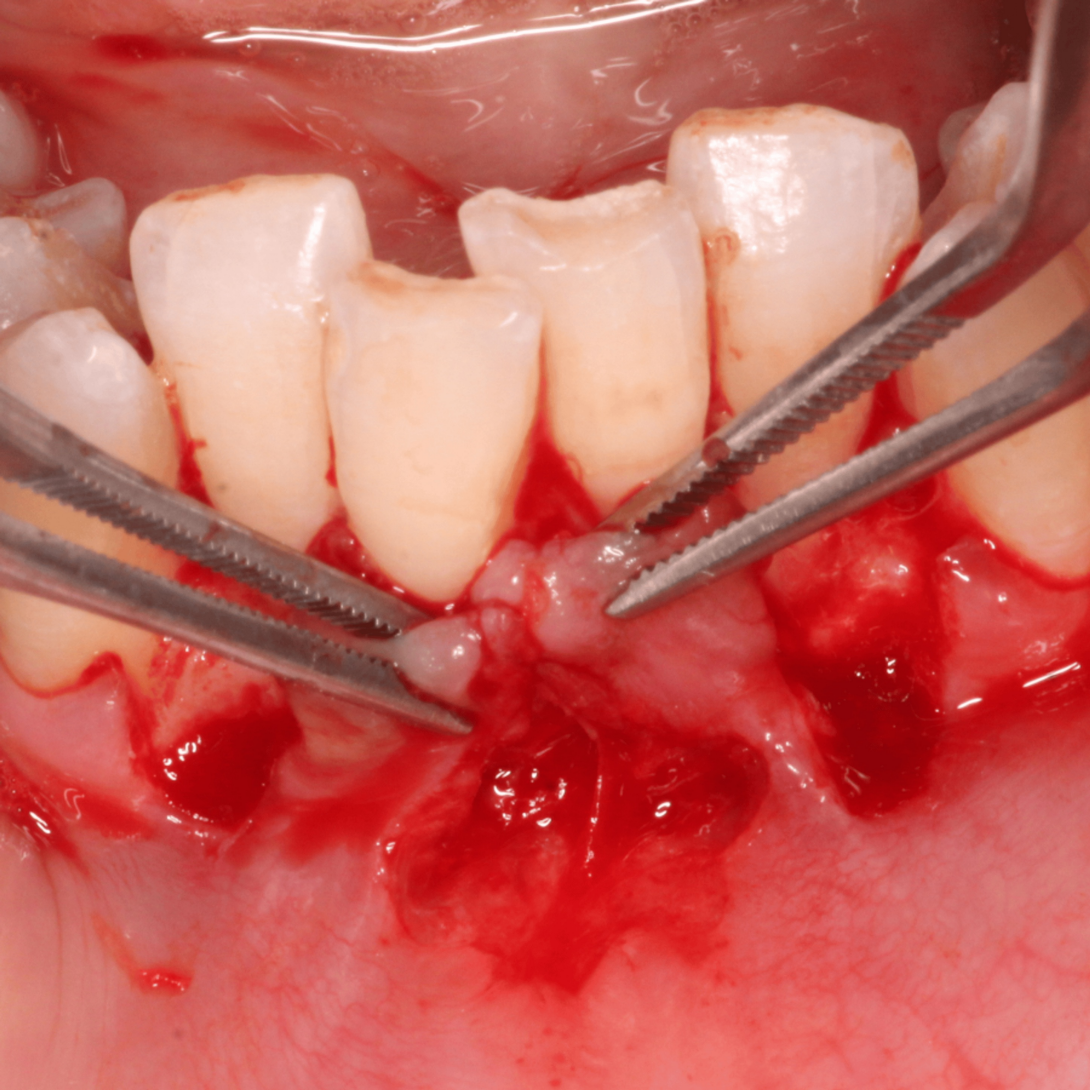
Approximation of the pedicle graft at recipient site using tissue forceps

**Figure 7:**
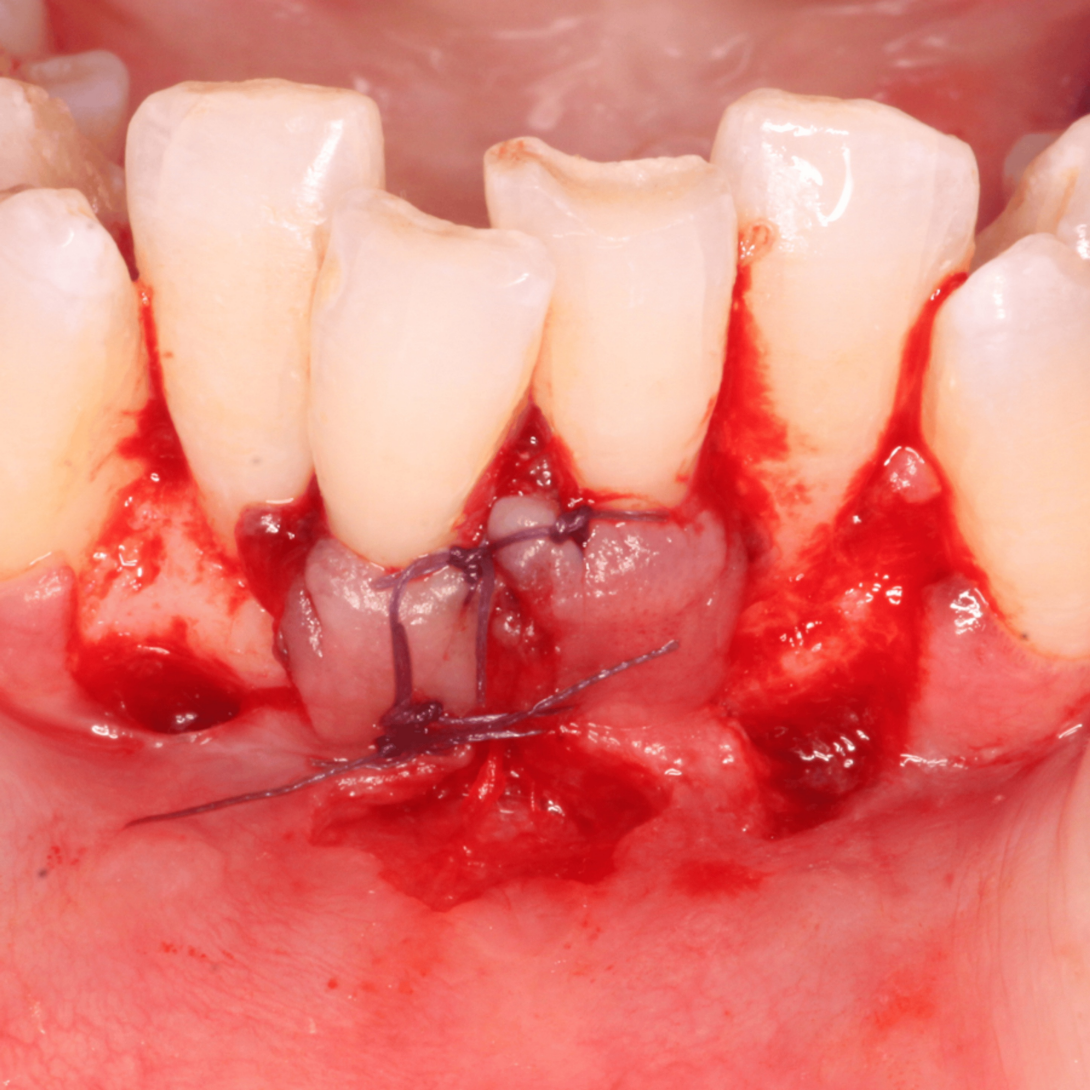
Precise joining of tissue edges with 5-0 Vicryl suture

**Figure 8:**
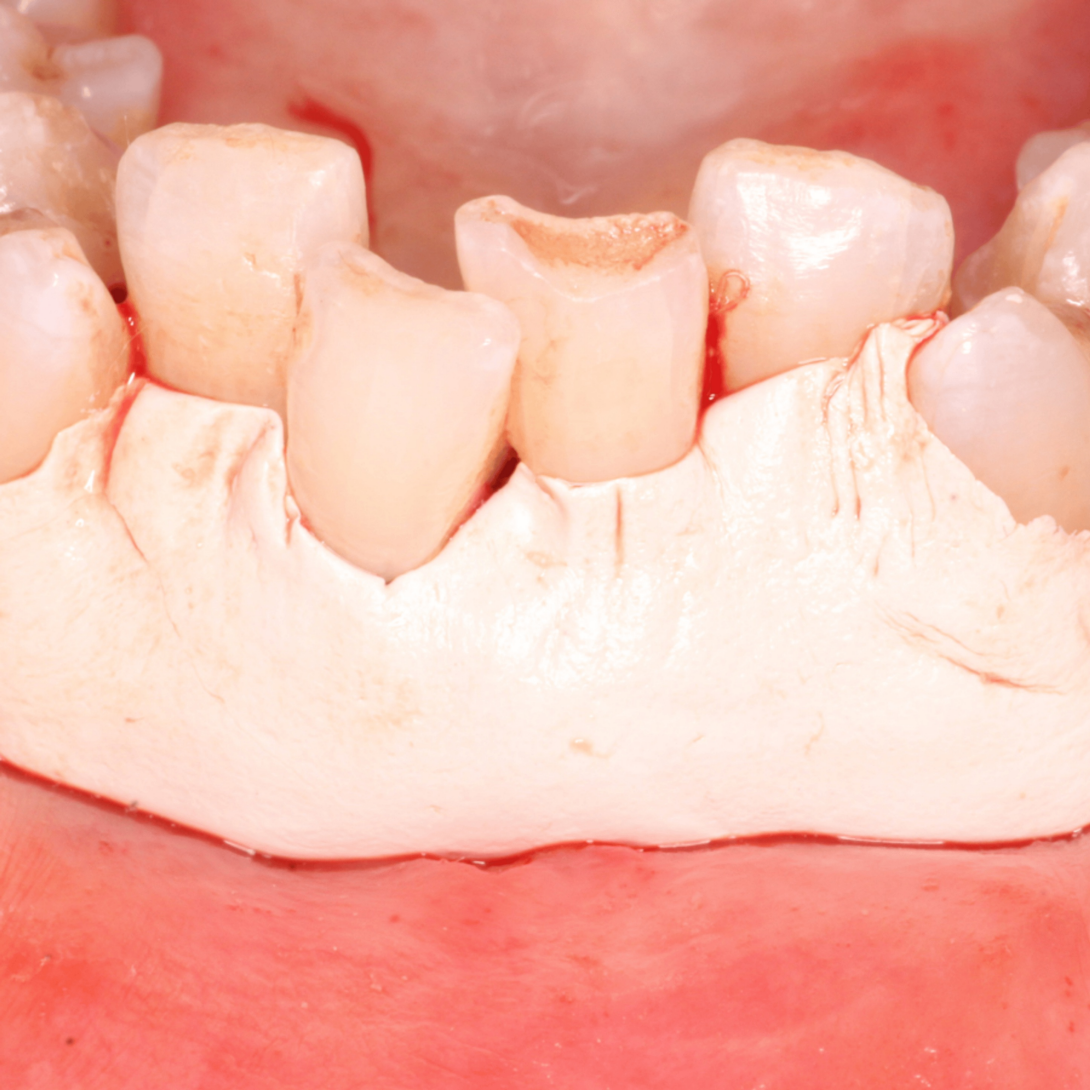
Coe-Pack given

The planned surgical flap configuration has been illustrated (Figure 3). After administering local anesthesia with 2% lidocaine, the recipient site was prepared using a No. 15 blade. Tissue deepithelialization was performed, accompanied by two vertical releasing incisions to facilitate surgical access (Figure 4). An intracrevicular incision was made along the bottom of the crevice, followed by two vertical incisions extending beyond the mucogingival junction at the line angles of the adjacent teeth, including the interdental papillae (Figure 5). A full-thickness pedicle flap was reflected, and the flap was passively positioned over the recipient site without tension using tissue forceps (Figure 6). The exposed root surface was gently planed using Gracey curettes. The pedicle flap was positioned, and a precise joining of the tissue edges was made using 5-0 Vicryl suture by sling suturing technique (Figure 7). A periodontal dressing (Coe-Pak) was applied to the surgical site to shield it from external irritants and facilitate healing (Figure 8).

The patient was given instructions to rinse with 0.2% chlorhexidine antidiscoloration system (ADS) mouthwash twice daily. The patient was instructed to refrain from brushing the surgical site and to avoid any trauma or pressure in the treated area. She was advised to not spit for 24 hours and to consume a soft diet for one week with no biting from the treated side. Antibiotics (amoxicillin 500 mg, administered three times daily for five days) and analgesics (aceclofenac 100 mg and paracetamol 500 mg, combined with aceclofenac-p, taken three times daily for five days) were prescribed. 

**Figure 9:**
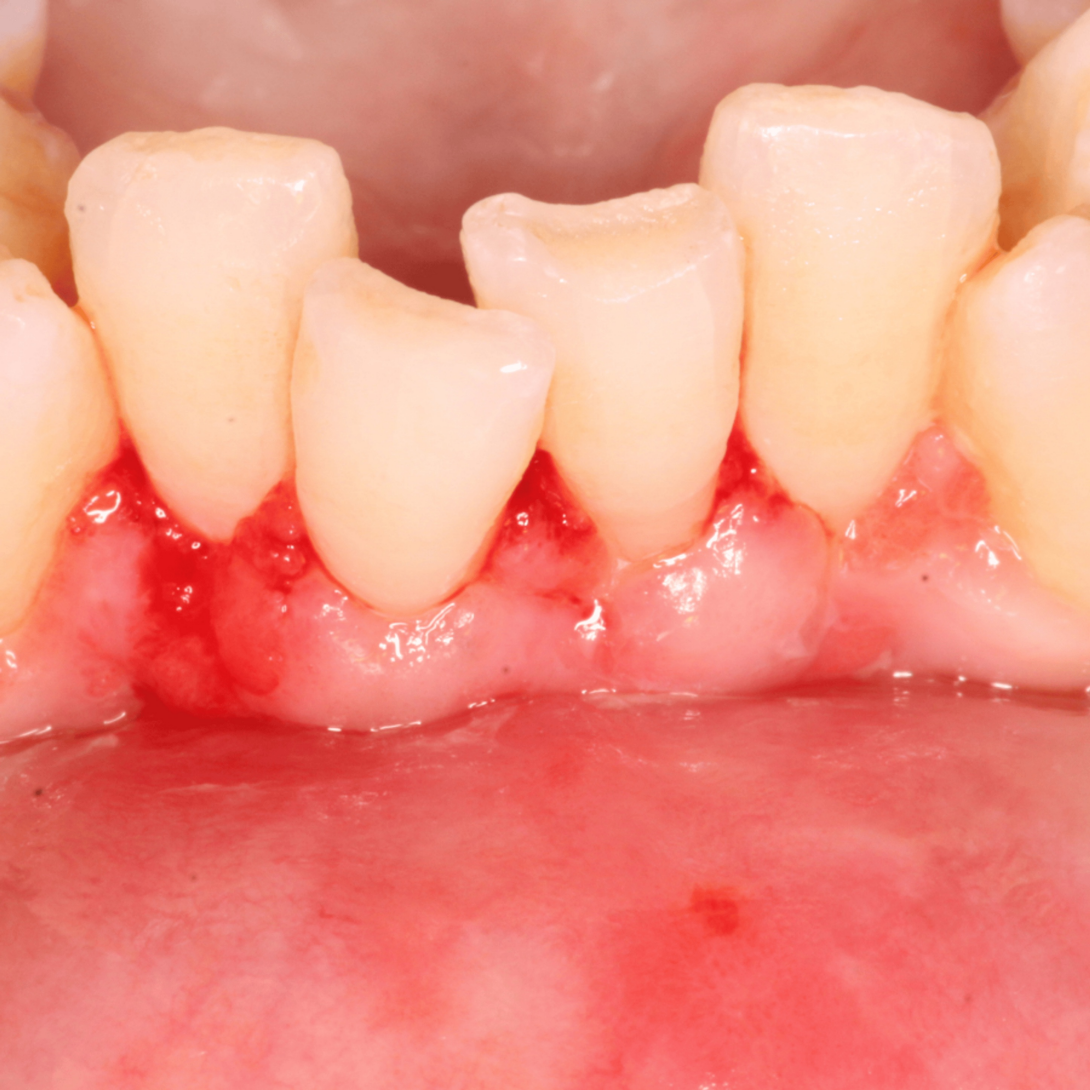
10 days follow-up

**Figure 10:**
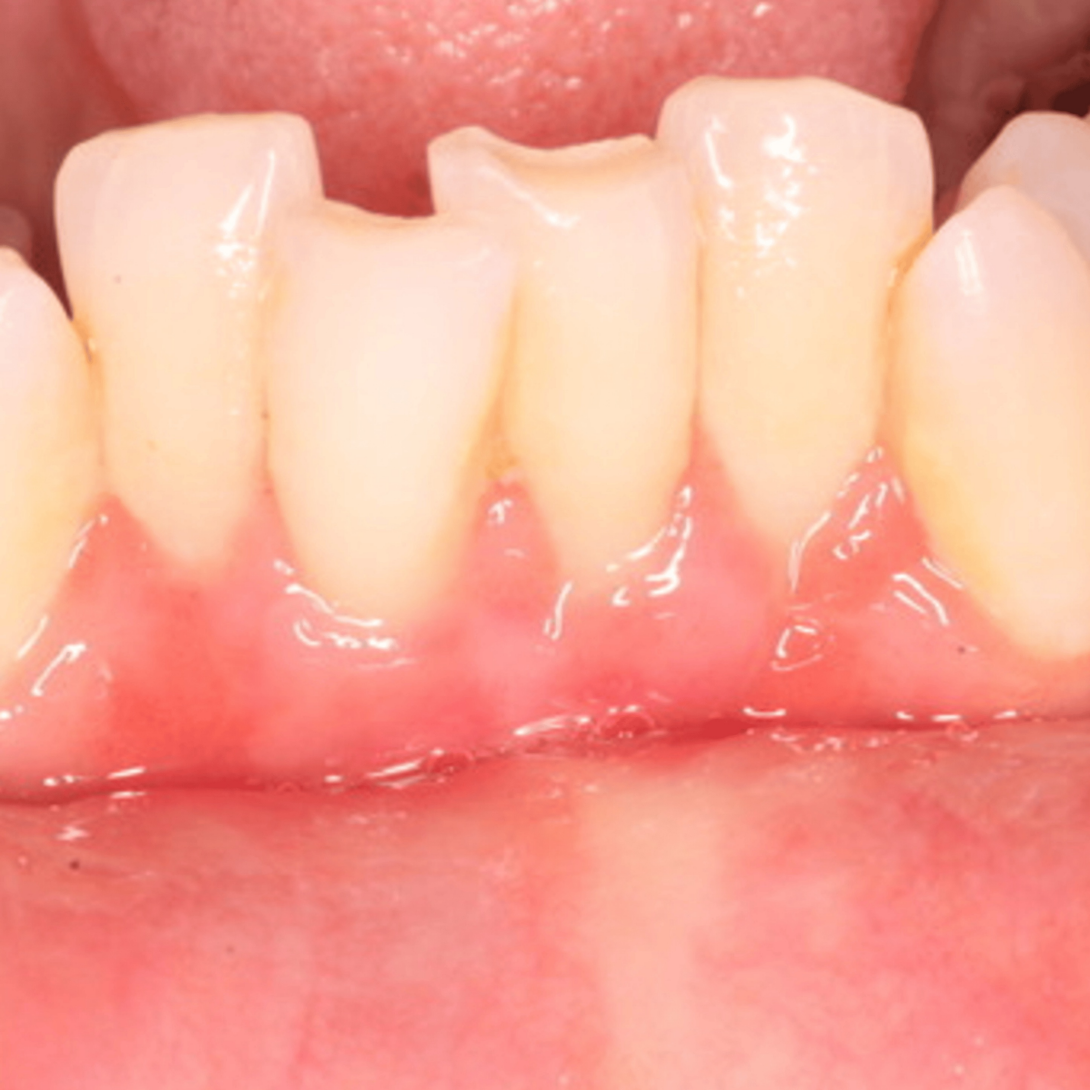
One month follow-up

The patient was scheduled for a follow-up appointment 10 days later for suture removal (Figure 9). The patient was enrolled in a maintenance program, which included professional plaque control and oral hygiene instructions. She was advised to resume mechanical tooth brushing with a soft toothbrush using the roll technique after two weeks. The patient was recalled back again after one month for reevaluation of maintenance (Figure 10).

**Figure 11:**
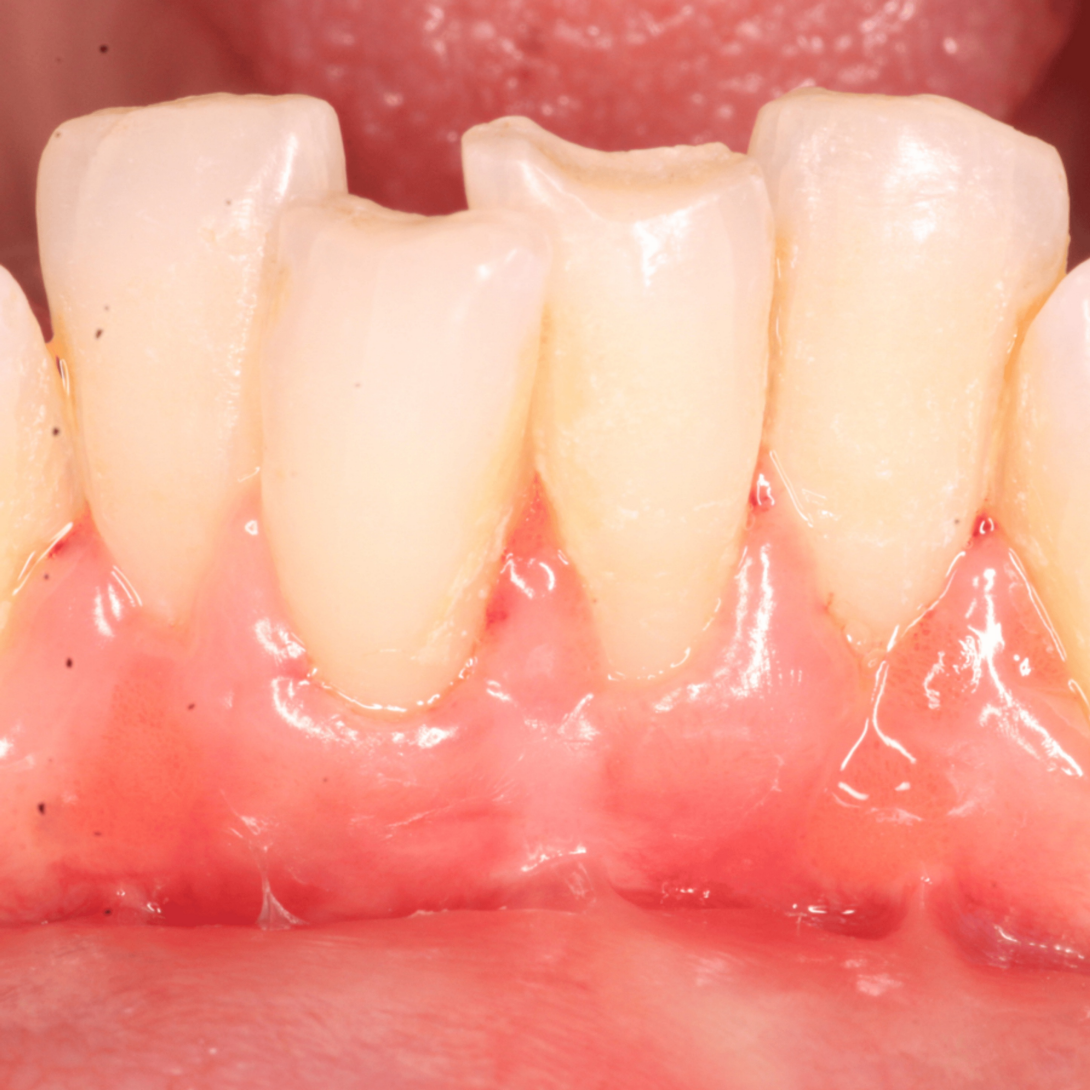
Three-month follow-up

**Figure 12:**
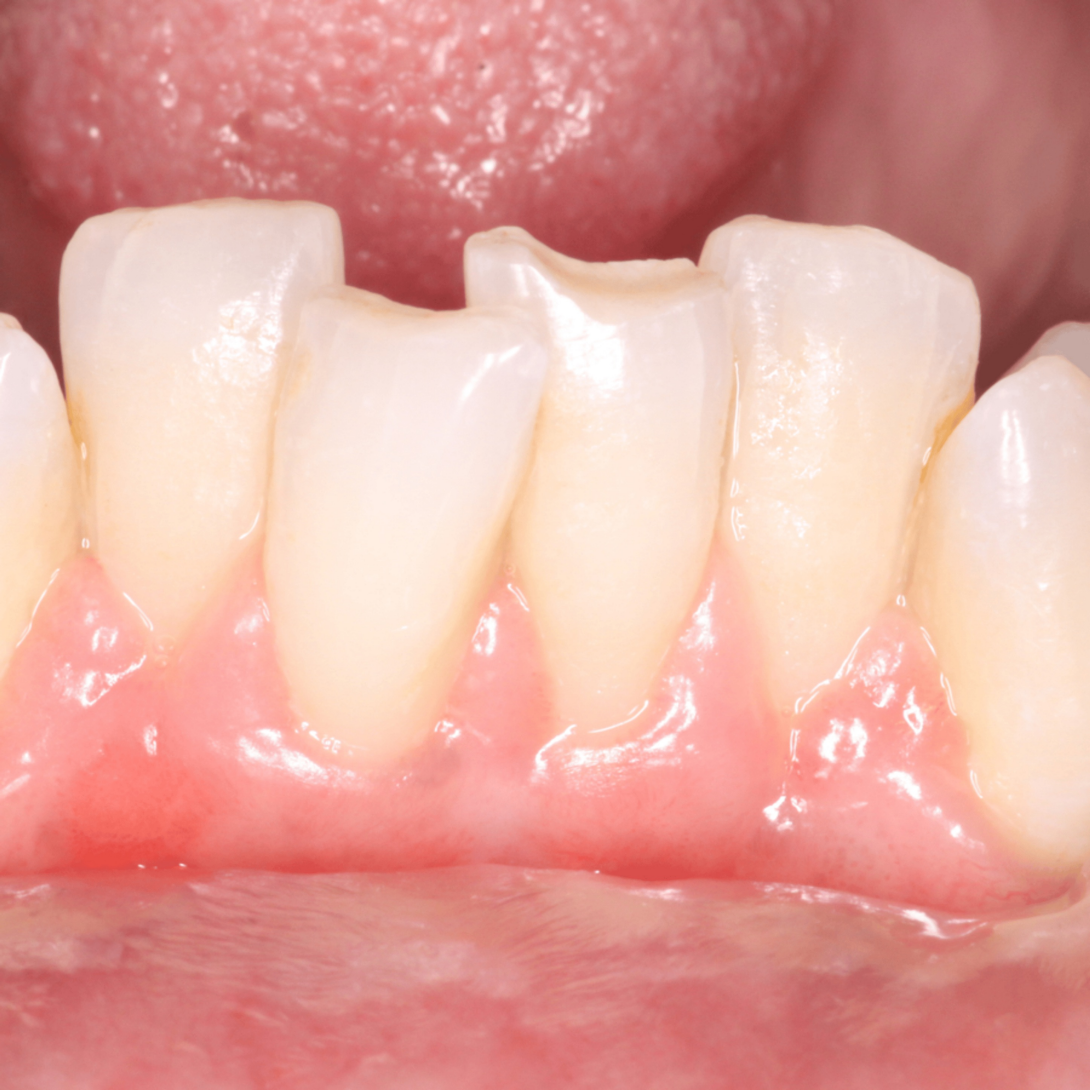
Six-month follow-up

The patient’s chief complaint of receding gum and dentinal hypersensitivity had resolved during the six-month follow-up (Figures 11, 12). She had adhered to all the postoperative oral hygiene instructions. 

## Discussion

Gingival recession often leads to aesthetic or sensitivity issues, necessitating root coverage procedures. A variety of techniques, including free gingival grafts, free connective tissue grafts, pedicle flaps, allografts, and guided tissue regeneration, have been proposed for root coverage. The selection of the optimal procedure is determined by factors such as the extent of gingival recession, the width of the attached gingiva, the number of teeth involved, and the desired aesthetic outcome. Each technique has its own specific indications and limitations. While free autogenous grafts provide reliable coverage, they require a second surgical site and are associated with postoperative patient discomfort. In contrast, lateral pedicle flaps benefit from a robust blood supply and eliminate the need for a second surgical site, thereby reducing patient morbidity.

The double papilla repositioned flap technique is particularly effective for the coverage of labial or lingual gingival recession, provided that the interdental papillae on either side of the denuded area remain intact [3]. A gingival recession of this nature is typically observed in areas exposed to mechanical trauma from improper tooth brushing leading to gingival destruction and formation of cleft. This pattern of recession is typically observed on the labial or buccal surfaces of teeth that are positioned labially relative to adjacent teeth. Also, prominent muscular or frenular attachments may contribute to the formation or persistence of these clefts.

Cohen and Ross in 1968 first proposed the partial thickness double papilla pedicle graft technique [3]. Subsequently, several modifications were introduced by various authors. In 1987, Nelson proposed a technique that combined a free connective tissue graft with a full-thickness double papilla graft [4]. In 1994, Harris further refined the technique by proposing the use of a partial-thickness double pedicle flap as an alternative to the full-thickness approach [5]. The double laterally rotated bilayer flap, another modification of the double papilla flap, has also demonstrated successful complete root coverage [6]. The newer technique of double papillae laterally positioned flap technique was combined with alloderm [7] not only provided complete root coverage but also resulted in an increase in the width of the keratinized attached gingiva.

This case needed adequate width of the attached gingiva as well as root coverage for restoring the periodontium. The adjacent thickness of the interdental papilla and healthy periodontium gave us a good indication of the double pedicle flap technique. This technique mitigates the patient discomfort and morbidity commonly associated with grafting procedures utilizing palatal donor sites. At the six-month follow-up, complete root coverage was seen along with an increase in the width of the keratinized tissue.

The present case exhibited comparable outcomes to those reported in previous research. In 2015, Acunzo et al. [8] conducted a study that demonstrated 88% root coverage along with an increase in keratinized tissue following the double papilla flap procedure for isolated tooth defects; similar outcomes were reported in a study conducted by Manisundar et al. in 2014 [9].

Additional procedures may be used in conjunction with double papilla for enhanced outcomes. In 2003, Tan and Ming [10] treated a recession defect using a connective tissue graft combined with the double papilla flap technique. The results demonstrated successful root coverage along with a notable increase in the amount of keratinized tissue (3 mm versus 1.8 mm).

This technique minimizes patient discomfort and complications commonly associated with grafting procedures that require palatal donor sites. It offers a time-efficient, minimally invasive, and aesthetically pleasing approach for treating isolated recession defects. The main disadvantage of this technique lies in the technical skill required to accurately join two small flaps in a manner that allows them to function as a single unified flap [3].

## Conclusions

This technique provides predictable aesthetic root coverage, with dual blood supply and optimal color integration with adjacent tissues, while also mitigating dentinal hypersensitivity. One of the significant advantages of this approach is that it eliminates the need for a second surgical site, thereby reducing patient discomfort and recovery time.
